# Enabling 3D electrical stimulation of adipose-derived decellularized extracellular matrix and reduced graphene oxide scaffolds *in vitro* using graphene electrodes

**DOI:** 10.1039/d5ra02570b

**Published:** 2025-09-01

**Authors:** Patrícia Alexandra Martins, Nathalie Barroca, Sandra I. Vieira, Bárbara M. de Sousa, Guilherme Gil, Mónica Cicuéndez, Laura Casarrubios, María José Feito, Rosalía Diez-Orejas, Maria Teresa Portolés, Bruno Figueiredo, Rui Silva, Andrea Garcia-Lizarribar, Pedro Fonseca, Luís Nero Alves, Paula A. A. P. Marques

**Affiliations:** a Centre for Mechanical Technology Automation (TEMA), Department of Mechanical Engineering, University of Aveiro Aveiro Portugal nbarroca@ua.pt paulam@ua.pt; b Department of Electronics, Telecommunications and Informatics, University of Aveiro Aveiro Portugal; c Instituto de Telecomunicações Aveiro Portugal; d Department of Medical Sciences, Institute of Biomedicine (iBiMED), University of Aveiro Aveiro Portugal; e Departamento de Química en Ciencias Farmacéuticas, Facultad de Farmacia, Universidad Complutense de Madrid (UCM), Instituto de Investigación Sanitaria del Hospital Clínico San Carlos (IdISSC) 28040 Madrid Spain; f Departamento de Bioquímica y Biología Molecular, Facultad de Ciencias Químicas, Universidad Complutense de Madrid (UCM), Instituto de Investigación Sanitaria del Hospital Clínico San Carlos (IdISSC) 28040 Madrid Spain; g Departamento de Microbiología y Parasitología, Facultad de Farmacia, Universidad Complutense de Madrid (UCM), Instituto de Investigación Sanitaria del Hospital Clínico San Carlos (IdISSC) 28040 Madrid Spain; h CIBER de Bioingeniería, Biomateriales y Nanomedicina, CIBER-BBN, ISCIII 28040 Madrid Spain; i Graphenest SA Sever do Vouga Portugal; j TECNALIA Basque Research and Technology Alliance (BRTA) San Sebastian Spain

## Abstract

Notwithstanding the demonstrated benefits of electrical stimulation in enhancing tissue functionality, existing state-of-the-art electrostimulation systems often depend on invasive electrodes or planar designs. This work exploits the versatility of graphene to fabricate biocompatible electrodes for the three-dimensional *in vitro* electrical stimulation of neural stem cells. A conductive green graphene-based ink was formulated and screen-printed as the bottom and top electrodes in a bottom-less standard culture well plate. Upon exposure to macrophages, although some oxidative stress was observed, this graphene-based ink did not elicit an increase in the pro-inflammatory cytokine IL-6. An analysis of the electrode impedance as a function of time and frequency was performed to optimize the 3D electrical stimulation. The efficacy of these graphene electrodes for electrically stimulating cells across 3D environments was investigated in scaffolds composed of a decellularized extracellular matrix and reduced graphene oxide, which had previously shown the capability to facilitate neuronal differentiation *in vitro* and to create a pro-regenerative microenvironment *in vivo*. Neural stem cells were seeded on these scaffolds and electrically stimulated with a 10 Hz bidirectional current signal of 200 μA for 1 hour daily. At the target frequency of 10 Hz, deemed advantageous for neural regeneration, a scaffold impedance below 800 Ω was ensured. The low-frequency 3D stimulation proved to enhance cellular mechanisms essential for the development of neuronal networks, including neuronal differentiation, neuritogenesis and neurite growth.

## Abstract 2

This work introduces scalable, non-invasive and cytocompatible graphene-based electrodes to enhance neuronal differentiation while preserving scaffold integrity *via* 3D electrical stimulation.

## Graphical abstract

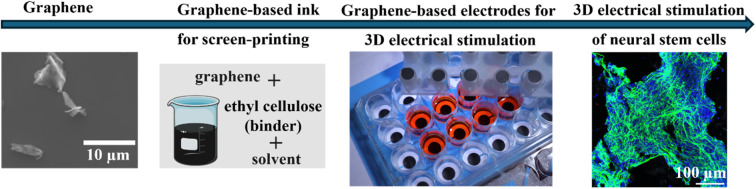

## Introduction

Three-dimensional (3D) constructs have been crucial in tissue engineering, disease modelling, and drug discovery. In neural tissue engineering, scaffolds are 3D environments conceptualized to enhance cell attachment, migration, and differentiation, promoting the formation of neural networks and tissue-like structures.^1^ In previous studies, we developed 3D scaffolds based on the integration of a decellularized extracellular matrix from the adipose tissue (adECM) and reduced graphene oxide (rGO) in view of repairing the injured spinal cord.^2,3^ Specifically, adECM has been shaped into 3D microenvironments using several biofabrication techniques such as ice templating,^2^ electrospinning combined with gas foaming,^3^ which consistently showed *in vitro* cytocompatibility with neural stem cells. When supplementing the adECM with rGO, the neuronal differentiation was significantly boosted *in vitro*.^2,3^ When implanted after a laminectomy for 6 weeks, the scaffolds created a pro-regenerative and angiogenic microenvironment.^4^ Further testing in a hemisection model revealed several encouraging outcomes, including tissue restoration, enhanced angiogenesis, the presence of myelinated axons, increased axonal growth at the host–scaffold interface, and unchanged astrocyte reactivity.^5^ Further additional cues can be delivered to these platforms to boost tissue repair. Electrical stimulation (ES) has shown promising evidence in modulating cellular behaviour in tissue engineering across several tissues.^6^*In vitro* ES has shown to induce galvanotaxis, activate signalling pathways and trigger neurotrophic release.^7^*In vivo*, ES is increasingly recognized as a standard approach in recovery, rehabilitation, and pain relief. For instance, deep brain stimulation can improve motor symptoms in Parkinson's disease^8^ and enhance the activation of neurons to promote signalling pathways for improved regeneration. In the peripheral nervous system, chronic compressive neuropathy, digital nerve transection, Bell's palsy and spinal accessory nerve damage have benefitted of low frequency ES (around 20 Hz), which resulted in improvements in sensory and motor functions.^9^ In the context of spinal cord injury (SCI) recovery, the objectives of ES extend beyond neural interaction. This therapeutic modality is one of the few that has been successfully translated from basic science to clinical applications. The documented mechanisms are related to the promotion of axonal outgrowth and plasticity both when using ES at injury site as well as in motor cortex.^10^ While ionic movement is typically linked to electrical signals of lower frequencies – typically below 100 Hz, neural activation is associated with higher frequencies, typically ranging from 1–10 kHz.^7–11^ Both approaches can promote neural regeneration and have been used both *in vitro* and *in vivo*. Also, by influencing ion movement in the target area, ES can alter the inflammatory environment, creating conditions more conducive to axonal regrowth and reducing inhibitory factors.^12^ Regarding *in vitro* assays, distinct devices have been developed in recent years for both ES and recording. Most designs apply ES in a two-dimensional (2D) manner, whereas others prioritise the recording of electrical signals. Examples of those recording devices are produced by companies like MultiChannel Systems MCS GmbH, Creative Bioarray, NMI Technologie Transfer GmbH and Axion Biosystems, Inc. Some solutions have been proposed for ES in a 3D manner,^13,14^ however not considering gravity effects or, sometimes, being invasive with the risk of puncturing the scaffold and affect its primary form.

In this work, we propose graphene-based electrodes that can be mounted in an easy and affordable manner in standard culture well plate to allow a 3D-stimulation to better mimic the 3D microenvironment in which cells operate. In recent years, graphene-based materials (GBM) have evolved far beyond their role as building blocks for nanomaterial-based scaffolds in tissue engineering.^15^ They now serve as the foundation for technological therapeutics, enabling high-precision and high-resolution neural interfacing. Their exceptional electrical and electrochemical properties, combined with their compatibility with flexible devices, make them ideal for applications in cortical electrodes, retinal and deep brain stimulation implants, and spinal cord stimulators.^16–18^ Although GBM have the potential for high conductivity (>10^5^ S m^−1^, in a multilayer approach),^19^ their actual conductivity is primarily determined by factors such as the fabrication process, purity, dimensions, and the presence of defects.^16^ Likewise, their biocompatibility is influenced by factors such as size, reduction degree, functionalisation, and sterilisation.^20^ Here, a graphene-based formulation was developed to achieve a green and conductive ink that can be processed *via* screen printing to produce biocompatible electrodes, which were further characterized in terms of performance, and macrophage response.

Stemming from our previous promising *in vitro* and *in vivo* evidence of the potential of adECM and rGO based scaffolds for SCI, the present study aims at probing the here-developed 3D electrical simulation device and investigating the effect of electrically stimulating a neural stem cell (NSCs) culture within these scaffolds in a 3D manner. The transplantation of NSC, either alone or in combination with other cells, has been demonstrated to be both feasible and safe in multiple completed clinical trials for patients with spinal cord injury.^21,22^ Among various stem cell types, NSCs have shown the most promising outcomes in terms of improving patients' motor and sensory scores.^21^ Further, the use of scaffolds to support transplanted NSCs is expected to enhance the efficacy of cell therapy. This combined approach has been investigated in pre-clinical studies^23^ and in clinical trials, including the transplantation of the NeuroRegen scaffold™ with NSCs (ClinicalTrials.gov ID (http://clinicaltrials.gov/): NCT02688049). In light of the clinical relevance of NSCs, an immortalized NSC line was seeded onto the scaffolds and electrically stimulated daily using the developed graphene-based electrodes mounted platform. NSCs proliferation and differentiation were evaluated as specific parameters to unveil the effects of the 3D ES with these new graphene-based electrodes.

## Results and discussion

### Graphene synthesis

**Fig. 1:**
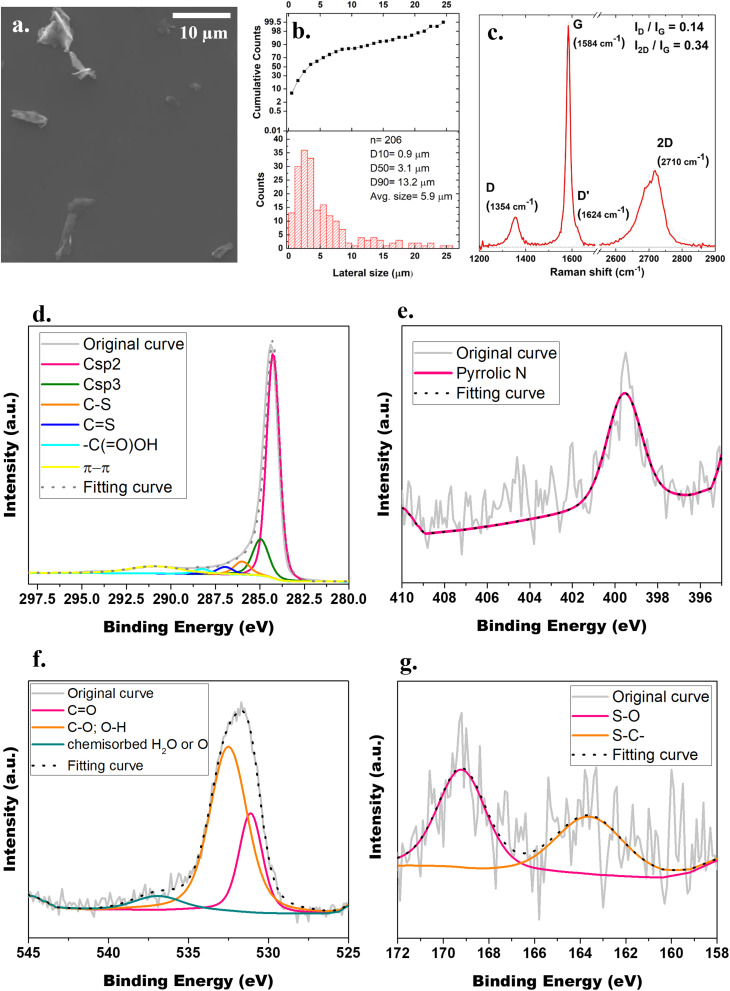
Graphene characterization: (a) graphene morphology using SEM; (b) graphene particle size distribution determined measuring over 200 individual flakes; (c) Raman analysis; (d)–(g) XPS spectra of (d) C1s, (e) N1s, (f) O1s and (g) S2p.

Graphene was prepared by a proprietary liquid-phase exfoliation process using ultrasonic cavitation and high-shear mixing. The resulting graphene particles, shown in Fig. 1a, were imaged using a high-vacuum SEM equipped with a secondary electron detector. Particle sizes were measured from the images using image analysis software, based on over 200 individual flakes. The particle size distribution, characterized by a *D*_10_ of 0.9 μm, a *D*_50_ of 3.1 μm, and a *D*_90_ of 13.2 μm, indicates a broad and right-skewed distribution, with most graphene flakes being below 5 μm and a smaller fraction extending up to 25 μm (Fig. 1b). Raman spectroscopy confirmed the presence of the three typical bands of carbon-based materials, specifically the band D (at 1354 cm^−1^) – indicating the presence of sp^3^ hybridized carbon and therefore the degree of defects in the structure –, the band G (at 1584 cm^−1^) related to the sp^2^ hybridized carbon atoms, and 2D at 2710 cm^−1^, characteristic of graphite and providing information about the number of graphene sheets (Fig. 1c). *I*_2D_/*I*_G_ peak ratio is routinely used to assess the number of graphene sheets while *I*_D_/*I*_G_ peak ratio is used for evaluating the presence of structural defects. Here, *I*_D_/*I*_G_ and *I*_2D_/*I*_G_ peak ratios, about 0.14 and 0.34 respectively, indicated that the produced graphene had few structural defects and was composed by multilayers.^24^

X-ray photoelectron spectroscopy was performed to identify qualitative- and quantitatively the elemental composition and chemical state of the produced graphene platelets (Fig. 1d–g). Table 1 summarizes the analysis performed on the spectrum obtained for C1s, O1s, N1s and S2p. Carbon spectrum identified the graphite-like sp^2^ hybridized carbon at 284.36 eV, the sp^3^ hybridized carbon of the oxidized area of graphene at 285.06 eV, the C–S bond at 286.1 eV, the C=S positioned at 287.06 eV, the carboxyl –C(=O)OH group at 288.36 eV ^25^ and finally the π–π transition at 291 eV. The O1s spectrum revealed 3 peaks: one derived from C=O bonds at 531.11 eV, C–O and O–H bonds at 532.49 eV and chemisorbed water or oxygen at 536.9 eV.^26^ Residual presence of N and S elements were also detected (0.33 and 0.09% respectively). *Via* deconvoluting of the S2p core-level spectrum, two peaks were identified as corresponding to polysulfide with terminal S (S–C–) at 163.61 eV and S–O at 169.19 eV. The N1s core-level line was quantified in one single position identified as the pyrrolic N at 399.56 eV.^25,26^

**Table 1:** XPS analysis of the elemental composition of graphene

Bond	Binding energy (eV)	Atomic (%)
Csp^2^	284.36	65.22
Csp^3^	285.06	13.61
C–S	286.10	4.87
C=S	287.06	2.71
–C(=O)OH	288.36	1.82
π–π	291.00	8.86
C=O	531.11	0.64
C–O; O–H	532.49	1.67
Chemisorbed H_2_O or O	536.90	0.18
Pyrrolic N	399.56	0.33
S–C–	163.61	0.04
S–O	169.19	0.05

### Graphene-based electrode system

**Fig. 2:**
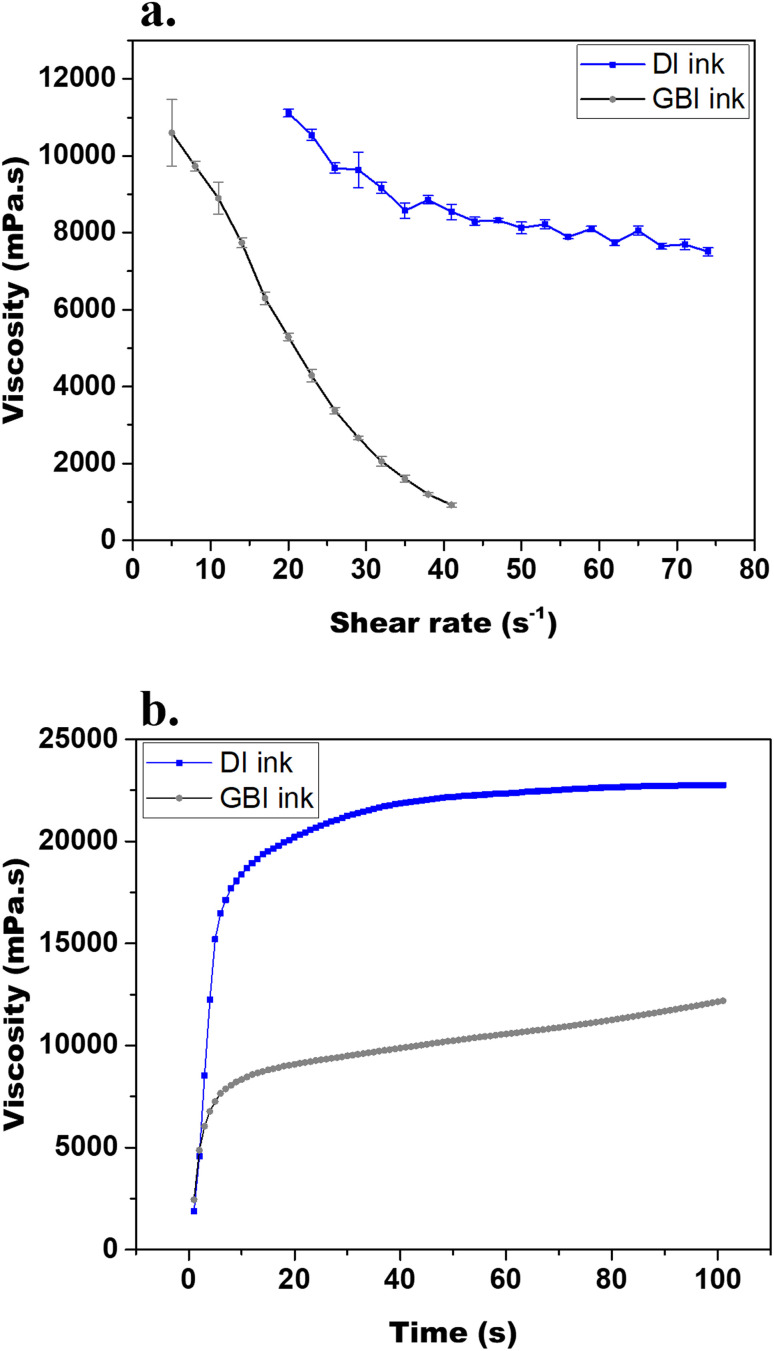
Rheological characterization of GBI and DI inks: (a) viscosity as a function of increasing shear rate; (b) viscosity as a function of time at a fixed shear rate of 5 s^−1^.

**Fig. 3:**
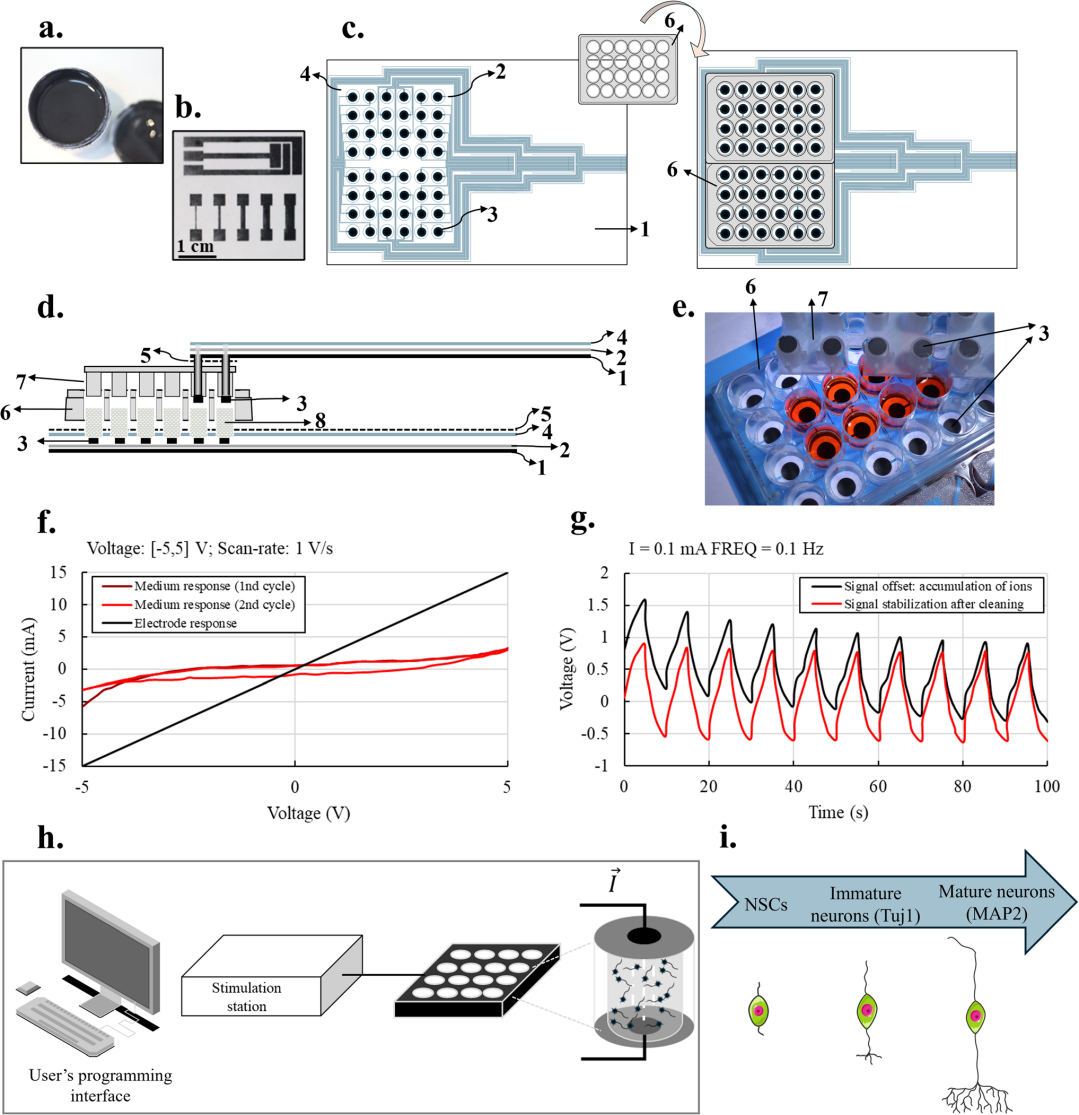
(a) Photograph of final graphene-based ink; (b) examples of several screen-printed designs including interdigitated electrodes; (c) schematic top view of the bottom electrodes and assembly with the bottomless well plate, (d) schematic representation of an embodiment of the device, namely a side view of the bottom electrode and lid electrode where: 1: polymeric sheet used as substrate for the GBI screen printed electrodes; 2: silver tracks layer; 3: screen-printed GBI electrodes layer that will be in direct contact with the scaffolds and the cell medium placed in each well; 4: screen-printed dielectric layer based on the commercial ink Sun Chemical D50706P3 (named DI); 5: double-sided adhesive; 6: bottomless wells; 7: lid including the inserts exhibiting the top GBI electrodes; 8: scaffolds; (e) photograph of the mounted device for 3D ES; (f) cyclic voltammetry (CV): current response for a CV cycle through top and bottom electrodes in dry contact (purposedly short-circuited); first and second CV cycle current response of EMEM stimulated through the electrodes; (g) regeneration properties after induced ionic accumulation on the electrode surface – square current input; (h) system overview for 3D ES of the scaffolds and seeded cells within the scaffolds and (i) scheme of studied markers along NSCs development.

The graphene-based ink was optimized based on the qualitative assessment of the printability by screen-printing and the adhesion to a polyethylene terephthalate (PET) foil. From varying solvent (ethanol/ethylene glycol, ethanol/terpineol, terpineol) as well as graphene and binder (ethyl-cellulose) concentrations (SI, Table S1), ethyl-cellulose above 1 wt% was identified as necessary to achieve adhesion and above 5 wt% to further promote an appropriate viscosity, graphene particles dispersion and good coverage. Concentrations of graphene above 12 wt% were favoured to obtain electrodes of low resistance. The selected ink formulation, named GBI, was characterized by a graphene:binder:solvent mass ratio of 4 : 1 : 20. The rheological behaviour of GBI was investigated by measuring the viscosity as a function of increasing shear rate (ranging from 5 to 74 s^−1^) (Fig. 2a) and at a fixed shear rate of 5 s^−1^ over time (Fig. 2b). A commercial ink from Sun Chemical (D50706P3), named DI in this work, was also characterized as a reference ink for screen-printing. This commercial ink was selected to screen-print a dielectric layer included in the device for 3D electrical stimulation. The rheological characterization of GBI reveals a strong shear-thinning profile, with a significant decrease in viscosity at increasing shear rates. This facilitates smooth flow during printing and supports uniform deposition. At a fixed shear rate over time, it suggests stability for prolonged processing. The combination of stable viscosity under constant shear and strong shear thinning behaviour under variable shear rates highlights the suitability of GBI ink for scalable printing processes such as screen printing and inkjet printing. This rheological profile not only enhances processability but also contributes to the functional performance of printed graphene layers by promoting uniform coverage and optimal adhesion to the surface of the substrate. Although its viscosity at high shear rates and recovery time are lower than those of the commercial DI ink, it was not expected to impair print quality at the targeted resolution. Indeed, GBI (Fig. 3a) enables to precisely screen-print different electrodes designs (Fig. 3b) spanning from the large circular shaped electrodes about 8 mm in diameter used for the 3D ES system to interdigitated electrodes and features as small as 250 μm without losing definition. Finally, at a shear rate of 5 s^−1^, GBI exibits a viscosity of 10 Pa s, similar to commercial inks such as Sericol Seridisc K from Fujifilm.^27^

The GBI electrodes were produced *via* 3 printing steps resulting in a dry thickness of 55 μm and a final sheet resistance of 200 ohms per square. The 3D ES device was developed to address the need of electrically stimulating cell cultures in a 3D manner, to better mimic the *in vivo* environment. The well plates for 3D ES were assembled by placing the polymeric sheet with the screen printed GBI electrodes on 2 bottomless 24-well plates (Fig. 3c) with medical grade double-sided adhesive (Fig. 3d and e). The silver screen printed layers, which must not contact with the culture (number 2 in Fig. 3c and d), are conductive paths for the current to flow from the stimulation station through the graphene-based electrodes and then through the scaffolds, placed inside the wells.

To better understand the electrical signal that was indeed applied to scaffolds and cells, both electrodes and scaffolds were characterized. Fig. 3f provides some evidence about the resistive behaviour of the electrodes and depicts a persistent ionic response of the medium when voltage cycles were applied. When the electrodes were short-circuited, a resistance of approximately 331.3 ± 0.1 Ω was obtained. In this regard, it is essential to know the dimensions of the wells, scaffolds and the amount of medium added during the culture as well as their electrical response to predict the electric power delivered to cells. The medium ionic accumulation on the electrodes, well-known as electrical double layer, is the effect of ionic movements induced by the electric field and it is represented in Fig. 3g. This effect makes a cleaning step necessary to regenerate the electrodes. The cleaning can be performed by applying electrical signals that promote the balance of ions or physically cleaning them with a liquid (*e.g.*, distilled pure water). Contrary to similar commercial applications, such as C-dish™ carbon electrode board from IonOptix (Milton, MA), the developed GBI electrodes do not need to be removed to be cleaned during cell culture. Noteworthy, the temperature of the culture environment was continuously monitored during the electrical stimulation experiments. No detectable temperature increase was observed during stimulation at 10 Hz, indicating that the graphene-based electrodes did not generate significant heat under these conditions. This observation is consistent with the well-established thermal conductivity of graphene-based materials, which facilitates rapid heat dissipation and minimizes local heating effects.

Finally, both top and bottom electrodes are connected to a stimulation station (described in SI: Table S2 and Fig. S2, S3) designed to enable the independent stimulation of 3 groups of 16 pair of electrodes (bottom and top) (see the organized 3 groups of 16 tracks each in Fig. 3c, S4 and S5). The user programming interface is connected to the stimulation station through a USB cable or Wi-Fi, providing the signal parameters for the stimulation and acquiring the voltage information at the terminals of each scaffold. In each of the wells, culture medium and a scaffold can be placed to be electrically stimulated (Fig. 3h). This concept was included in an international patent.^28^ NSCs were seeded on the scaffolds and electrically stimulated for 14 days (bidirectional current signal of 200 μA, 10 Hz and 1 hour daily), during which proliferation and neuronal differentiation were analysed, specifically Tuj1 and MAP2 expression were used as markers of immature and mature neurons respectively (Fig. 3i).

### GBI biocompatibility and immune response

**Fig. 4:**
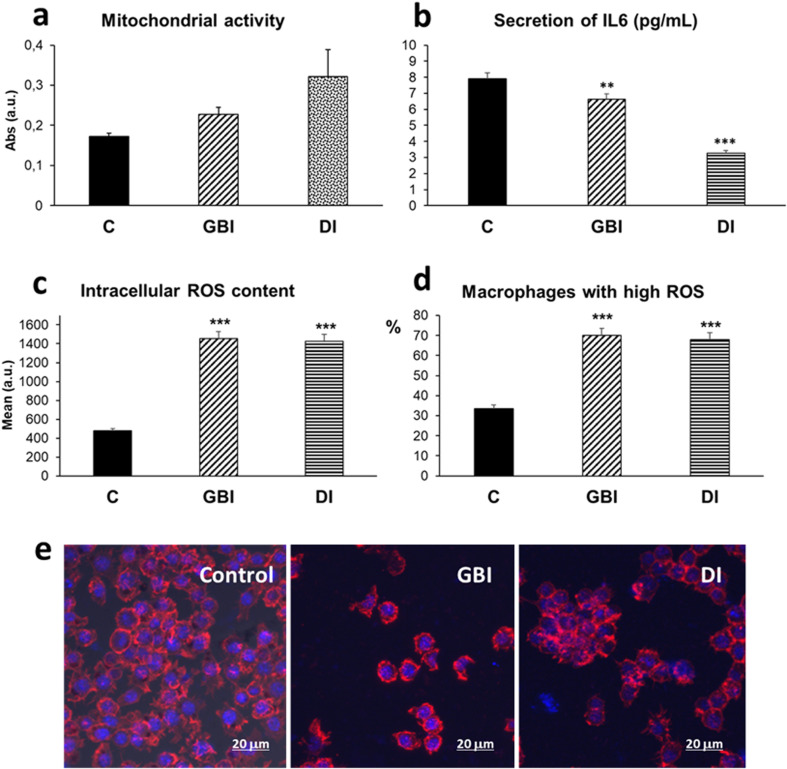
Response of RAW-264.7 macrophages cultured for 24 hours in direct contact with GBI and the commercial dielectric ink (DI). Control macrophages were cultured in parallel in the absence of these materials. (a) Mitochondrial activity; (b) secretion of interleukin 6 (IL-6); (c) intracellular reactive oxygen species (ROS) content; (d) percentage of macrophages with high ROS content; (e) confocal microscopy images showing control macrophages and macrophages cultured in contact with GBI and DI inks. The actin filaments of the cytoskeleton were stained with rhodamine-phalloidin (red) and cell nuclei with DAPI (blue). Statistical significance (**p < 0.01), (***p < 0.005).

Because macrophages are directly involved in the innate immune response and can play both positive and negative roles in biomaterial integration and tissue repair processes,^29^ the biocompatibility of both GBI and the commercial ink (DI) used as dielectric coating was evaluated by assessing the response of RAW-264.7 macrophages cultured for 24 hours in direct contact with these inks. Tissue repair may be seriously affected by the immune response due to the macrophage plasticity between M1 pro-inflammatory and M2 reparative phenotypes, characterized by specific cell-surface markers and the secretion of different cytokines.^30^ Here, RAW 264.7 cells were selected due to their phagocytic and pinocytic capabilities, as well as their functional stability, which make them widely used for evaluating the innate immune response and potential cytotoxicity of biomaterials. It has been demonstrated that their phenotype—defined by the expression of macrophage-specific genes, surface markers, and functional properties such as phagocytosis and nitric oxide production—remains stable across multiple passages (up to passage number 30).^31^ Diverse techniques were employed to evaluate different parameters of the macrophage response to GBI and DI inks: macrophage mitochondrial activity, intracellular reactive oxygen species (ROS) content, percentage of macrophages with high ROS content, and cell morphology through the visualization of the actin cytoskeleton and the nucleus (Fig. 4). In addition, the secretion of interleukin 6 (IL-6) was determined as cytokine released by pro-inflammatory M1 macrophages. As it can be observed in Fig. 4a, GBI and DI inks induced significant increase of macrophage mitochondrial activity in comparison with control macrophages. This increased mitochondrial activity could be related to enhanced ROS production, since ROS are mainly generated by mitochondria. ROS production and elimination are tightly controlled inside the cells as increased ROS production can lead to oxidative stress, damaging mitochondrial proteins, membranes and DNA, as well as impairing the ability of mitochondria to synthesize ATP and numerous metabolic pathways.^32^ On the other hand, the production of ROS and the induction of oxidative stress is a key mechanism involved in the cytotoxicity of GBM.^33^

In the present study, GBI and DI inks induced significant increases in both macrophage intracellular ROS content and macrophage population with high ROS levels (Fig. 4c and d, respectively) evaluated by flow cytometry. Thus, both inks induced oxidative stress in macrophages, consistent with the effects characteristic of GBM mentioned above. Excessive ROS can lead to destruction of cellular components and ultimately to cell death *via* apoptosis or necrosis.^34^ Confocal studies evidenced the maintenance of the macrophage characteristic morphology after direct contact for 24 h with GBI and DI without signs of necrosis and apoptosis (Fig. 4e). Concerning their effect on the secretion of IL-6 by macrophages, these materials induced a significant IL-6 production decrease (Fig. 4b). This cytokine has generally been considered a pro-inflammatory agent, but there is increasing evidence that IL-6 is a pleiotropic cytokine with both pro- and anti-inflammatory effects and with wound healing ability.^35,36^ Previous studies with RAW-264.7 macrophages treated with graphene oxide nanostructures have evidenced a dose-dependent increase of this cytokine secretion induced by graphene oxide. However, lower levels of IL-6 than that in controls were obtained after reducing the graphene oxide.^37^ The obtained results could indicate an absence of pro-inflammatory effects of both the dielectric ink as well as the developed graphene-based ink but also a decreased functional capacity of macrophages to produce this cytokine in the presence of these materials. Because graphene cytocompatibility depends on several factors such as sheet dimensions, agglomeration and packing, the manufactured electrodes were also readily evaluated when contacting directly with NSCs. The metabolic activity of NSCs seeded on top of the electrode was probed *via* the resazurin assay for 11 days. The cells exhibited a remarkable affinity towards the screen-printed electrode, showing adhesion, spreading and proliferation into large clusters (Fig. S6a, black arrows), as corroborated by the measured elevated metabolic activity (Fig. S6b). Neurite-like projections are observed between cells and clusters as early as 11 days (Fig. S6c, white arrows). Whereas the screen-printed graphene electrodes were conceptualized for the stimulation of cells across 3D microenvironments such as scaffolds, this result suggests that the developed graphene-based electrodes have also the potential for neural stem cell culture in direct contact to study cell mechanisms under planar ES.

### *In vitro* proof of concept

We have conducted extensive work on developing scaffolds by interfacing graphene-based materials with decellularized extracellular matrix (dECM). In our initial studies, adipose tissue was explored as a source of dECM, and comprehensive proteomic and biochemical analyses revealed that porcine-derived adECM retains over 70% of native proteins. Additionally, macrophages cultured within adECM shifted towards a pro-reparative phenotype, indicating a positive immunomodulatory effect.^38^ The unique biochemistry of adECM also supports the adhesion and growth of neural stem cells and embryonic neural progenitor cells. We further demonstrated that adECM can act as a physical crosslinker for rGO, enabling the fabrication of composites containing up to 50 wt% rGO, which boosted neural stem cell differentiation into neurons.^2^ Building on these promising *in vitro* results, the scaffolds were subsequently evaluated *in vivo* in contact with the spinal cord. Following a laminectomy at the T10 vertebra, the scaffolds remained in place for six weeks and were infiltrated by macrophages predominantly polarized towards a pro-regenerative phenotype, with no adverse effects detected by organ-specific histopathology and biochemical analysis.^4^ In a subsequent T10 hemisection model, the scaffolds promoted a tissue-restorative and angiogenic response. Some functional recovery was observed, although limited, which was attributed to limited axonal sprouting.^5^ Together, these *in vitro* and *in vivo* studies highlight that these scaffolds are promising candidates to repair the injured spinal cord. Considering that these results were obtained in the absence of any cellular cue or additional stimulus such as electrical stimulation, it is hypothesized that more meaningful recovery could be obtained in the future using combinatorial therapies such as exploiting these scaffolds together with transplanted NSCs and electrical stimulation. This work aims to provide evidence that these scaffolds can be used to harbour neural stem cells and be suitable for 3D electrical stimulation.

### Scaffolds characterization

**Fig. 5:**
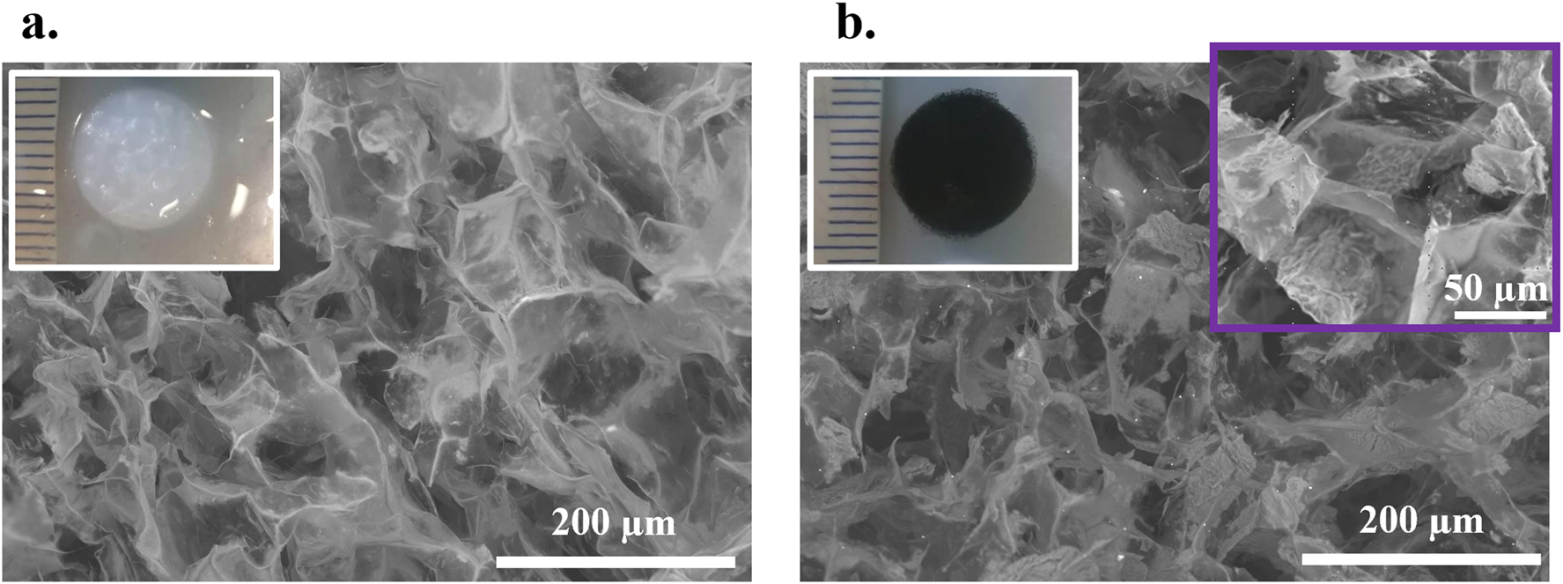
Morphological characterization of scaffolds: SEM micrographs and photographs of (a) adECM scaffolds and (b) adECM/rGO scaffolds.

A thorough characterization of these scaffolds can be found in previous publications from our group.^2,4,5^ Nonetheless, some morphological properties are shown in Fig. 5. Both formulations – adECM and adECM coupled with rGO – exhibited an open porous structure with a pore size permissive of NSCs ingrowth^2^ and a compressive Young's modulus in the order of 1 kPa, compliant with the soft tissue of the spinal cord.^39^

**Fig. 6:**
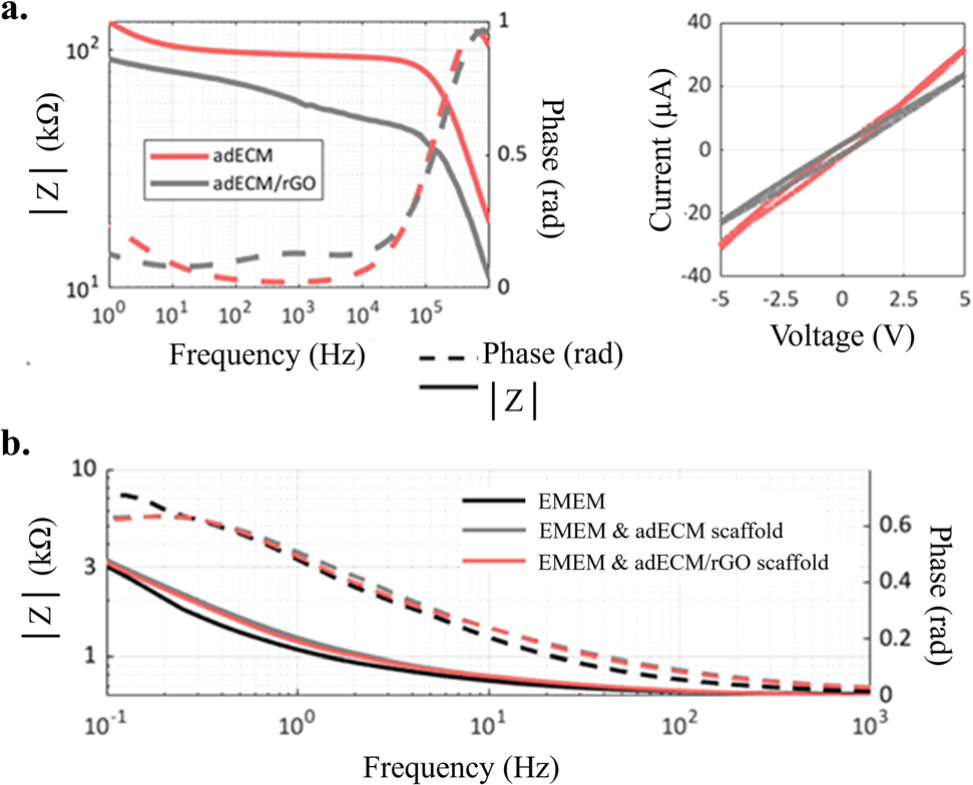
(a) Electrochemical Impedance Spectroscopy (EIS) results and cyclic voltammetry (CV) measurements of scaffolds in deionized water; (b) EIS (50 mV) for EMEM and EMEM with submersion of each scaffold, impedance, and phase. Measurements were conducted with the scaffolds placed in the well and then filled with the medium.

Electrochemical impedance spectroscopy (Fig. 6) revealed that while scaffolds containing rGO significantly reduce impedance in water (by 39.84 kΩ at 1 Hz and 41.47 kΩ at 10 kHz) (Fig. 6a), the high ionic conductivity of EMEM culture medium attenuates these differences, resulting in only marginally lower impedance for rGO-containing scaffolds (Fig. 6b). Previous analyses on these scaffolds using scanning spreading resistance microscopy revealed that adECM/rGO scaffolds did not exhibit a larger electrical conductivity than the adECM ones due to the moderate reduction of rGO, exploited in these scaffolds for providing structural stability to the highly loaded adECM/rGO scaffolds.^2^ Moreover, Raman-SNOM analysis previously detailed an increased presence of defects/disorders in this rGO that further curtails electrical conductivity.^40^ Therefore, it was not expected that the rGO-containing scaffolds would exhibit substantially lower impedance in EMEM, which has much higher electrical conductivity (15 mS cm^−1^)^7^ compared to distilled water (less than 0.01 mS cm^−1^)^41^ and therefore dominates the impedance behaviour of the scaffold-medium system.

In more details, at low frequencies, *i.e.* from 10^−1^ to 10 Hz, where polarization and double-layer effects are more pronounced, both scaffolds introduce slightly higher resistivity compared to the medium alone, reflecting interfacial interactions and partial ion pathway obstruction within the porous architecture. However, at higher frequencies, these differences diminish, and the impedance profiles converge, indicating that ionic conduction from the medium dominates in the culture environment. Interestingly, scaffolds with rGO exhibited a slightly higher resistivity than pure adECM scaffolds in this low-frequency regime within EMEM. This may be attributed to the high specific surface area of rGO and its reduced functional groups which are known to facilitate the adsorption of biomolecules. Here, a higher accumulation and adsorption of ions, proteins, or amino acids from the medium onto rGO lead to increased interfacial resistance, double layer capacitance and charge transfer resistance.^42,43^

Importantly, living tissue environments are also dominated by ionic conduction,^44^ and thus a scaffold that behaves predictably within a conductive medium while allowing safe, distributed electrical fields remains suitable for studying cell responses under electrical stimulation. Since the selected stimulation regime relies on a 10 Hz frequency, reported to support neural regeneration, the resulting scaffold impedance in EMEM falls between 743 and 789 Ω, which perfectly aligns with clinical impedance ranges for deep brain stimulation electrodes in patients (typically 500–1500 Ω).^45^ This confirms that, despite the limited contribution to overall conductivity of both scaffolds, they can effectively deliver electrical stimulation within a physiologically relevant impedance range, supporting their suitability for *in vitro* neuromodulation studies under clinically relevant conditions.

### 3D electrical stimulation of adECM and adECM/rGO scaffolds

**Fig. 7:**
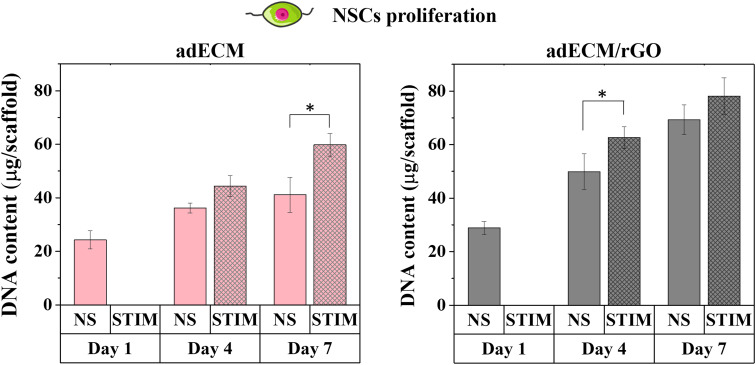
Proliferation of NSCs seeded on adECM and adECM/rGO scaffolds with and without 3D ES. Statistical significance indicated for comparison of non-stimulated and electrically stimulated scaffolds for each day (*p < 0.05).

While the combination of ES and conductive nanomaterials has been shown to enhance neurogenesis and NSCs differentiation^46,47^—primarily in planar experiments—there is a need to expand these strategies to include scaffolds made from other biomaterials, such as extracellular matrices that better replicate tissue environments. Additionally, stimulating these scaffolds in 3D configurations would provide more physiologically relevant insights. Measuring the DNA content per scaffold (Fig. 7), an indicator of NSCs proliferation, revealed that adECM scaffolds support steady but slow NSCs growth. However, when subjected to ES, a significant increase in proliferation was observed at 7 days of culture. Increased NSCs proliferation upon low frequency ES has been observed both when cultured directly on top of electrodes as well as on electrically conductive scaffolds.^46^ Although the adECM scaffold itself is not electrically conductive, electrical signalling is facilitated *in vitro* by the relatively high conductivity of the culture media (15 mS cm^−1^), which closely resembles the conductivity of extracellular fluid *in vivo* (3–12 mS cm^−1^).^7^ In the adECM/rGO scaffolds without stimulation, the incorporation of rGO significantly boosted the initial proliferation of NSCs, consistent with our previous findings on these scaffolds.^2^ While ES further augmented proliferation on day 4, the possible cumulative effect of rGO and ES became non-significant by day 7. This may be attributed to the presence of rGO, also known to encourage NSCs differentiation,^2,3,48,49^ therefore shifting cell mechanisms towards specialization and decreasing the proliferation rate by day 7. For the formation of neuronal circuits, neurite outgrowth and axonal elongation are key processes in neural tissue repair. Therefore, attention was directed towards understanding the effect of the 3D ES on events related to the differentiation of NSCs, particularly lineage commitment and neuritogenesis. For that purpose, the scaffolds were immunolabeled for β-tubulin III (Tuj1), the β-tubulin isoform of neurons, present in their cell bodies and neurites (dendrites and axons), and a commonly used marker of immature neurons. Further, since β-tubulin III is also expressed by fetal astrocytes, cells were also labelled for glial fibrillary acidic protein (GFAP), a specific astrocyte cytoskeletal marker.

**Fig. 8:**
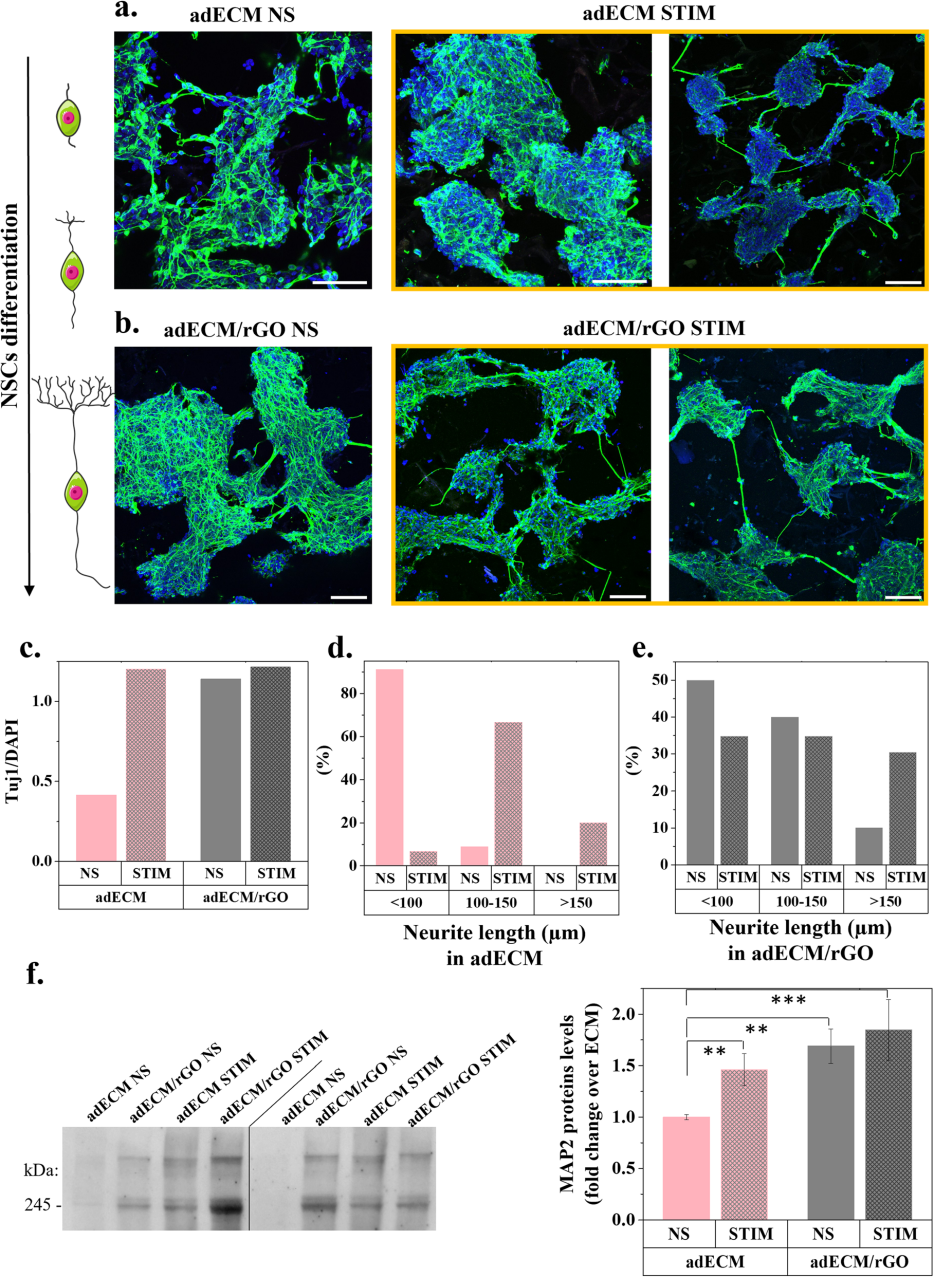
Differentiation of NSCs on adECM and adECM/rGO scaffolds 10 days after RA-induced differentiation: (a) confocal imaging – immunocytochemistry of neuronal (Tuj1, green), astrocyte markers (GFAP, red) and cell nuclei (DAPI, blue) – of the NSCs with (STIM) and without (NS) 3D ES on (a) adECM scaffolds and (b) adECM/rGO scaffolds; scale 100 μm; (c) semi-quantitative analysis of NSCs differentiation by Tuj1/DAPI area – one representative scaffold per condition was analysed, with 36 areas scanned along its cross-section to assess differentiation from the bottom to the top of the scaffold; (d) and (e) length of neurites branching out of cell clusters in adECM and adECM/rGO respectively; (f) immunoblot analysis of the levels of the mature neuronal marker MAP2 (*N* = 8), two here presented (lanes were not cropped nor rearranged). Statistical significance: (**p < 0.01), (***p < 0.005).

As shown in Fig. 8a and b, NSCs demonstrated wide immunoreactivity for Tuj1 (neuronal marker) while GFAP staining remained negligible for all scaffolds in both non stimulated and electrical stimulated conditions, thereby indicating that these scaffolds are permissive microenvironments for neuronal differentiation. In comparison to non-stimulated adECM scaffolds, confocal images revealed that electrically stimulated ones presented an intense labelling of thick neurite bundles connecting adjacent NSCs clusters. Within cell clusters, the development of a highly branched neuritic network is visible within the clusters, appreciably denser than on non-stimulated adECM scaffolds. Semi-quantitative analysis was conducted by evaluating the positive staining area of Tuj1 per DAPI (Fig. 8c) by analysing 36 areas scanned along each scaffold cross-section to assess differentiation from the bottom to the top of the scaffold. A 3-fold increase in the Tuj1/DAPI area was measured in electrically stimulated adECM scaffolds, translating a remarkably increased neuritogenesis. Also, while around 90% of neurites branching out of cell clusters in non-stimulated scaffolds were below 100 μm, ES greatly promoted neurite growth with more than 90% above 100 μm (Fig. 8d). Given that Tuj1 is an immature neuronal marker, the neuritogenic effects of the 3D ES during NSCs differentiation were further confirmed by quantitative immunoblot analysis of a mature neuronal marker, the microtubule-associated protein 2 (MAP2) (images of the full blots, uncropped and unprocessed are available in Fig. S7). Although some inter-experimental variability could be observed in the immunoblots (Fig. 8f, left), upon densitometric quantification and correction to a loading control (Ponceau S dye, in Fig. S8), increased neuritogenesis was evident in some groups. Indeed, immunoblot data (Fig. 8f, right) revealed that the levels of MAP2, a neuron-specific structural protein that stabilizes microtubules in the dendrites of postmitotic neurons, were significantly increased by approximately 1.69-fold in rGO-containing scaffolds, when compared to adECM scaffolds only. This was expected since our previous work demonstrated the differentiation-inducing effect of rGO.^2,3^ Notably, regarding the exogenous ES, a significant increase in MAP2 expression of about 1.46-fold was induced in the adECM scaffolds. Conversely, in rGO-containing scaffolds that previously demonstrated to induce differentiation and neuritogenesis, no statistically significant increase was observed upon stimulation. While literature reports that electrically stimulating conductive rGO-based scaffolds may lead to increased cell activity,^50^ this was not observed for the rGO-containing scaffolds presented here. Since electrochemical impedance spectroscopy revealed that these lack significant electrical conductivity on a macroscopic scale, this may account for the absence of cumulative effect when applying the stimulation. Nevertheless, when examining the length of neurites, specifically those extending from cell clusters, a 3-fold increase in the percentage of neurites exceeding 150 μm (Fig. 8e) was observed. Although ES in highly differentiation-inductive scaffolds like adECM/rGO did not significantly elevate Tuj1 and MAP2 markers, it did enhance the length of neurites connecting cell clusters. By promoting longer neurite bundles branching from neurospheres towards neighbouring clusters, ES likely facilitates improved neuronal communication throughout the scaffold.

Finally, whereas the emergence of a mature and well-organized neuronal network under ES is often documented on conductive substrates^51^ to optimize its efficacy, our findings corroborate that controlled exogenous electric fields significantly enhance cell responses, even in non-conductive scaffolds. This enhancement is particularly evident in the robust induction of neuritogenesis, observed longitudinally across the scaffold. Specifically, on adECM scaffolds lacking rGO, a remarkable neuritogenesis was observed following one hour of daily stimulation for 14 days. This study serves as a proof of concept, demonstrating that the proposed 3D non-invasive ES holds relevance across different systems.

## Conclusions

A device for the three-dimensional electrical stimulation of cells cultured within scaffolds was developed using a simple and affordable strategy based on graphene-based electrodes. This practical platform can be readily implemented without requiring complex or costly setups, facilitating the broader adoption of electrically stimulated 3D cultures in tissue engineering. A conductive and green graphene-based ink based on graphene/ethyl-cellulose/terpineol (mass ratio of 4 : 1 : 20) was screen-printed to make up for the biocompatible electrodes at the bottom and top of the culture wells. These screen-printed graphene-based electrodes, with an 8 mm diameter, demonstrated a dry short-circuited impedance of 331.3 ± 0.1 Ω and were cytocompatible in direct contact with neural stem cells. Moreover, in contact with macrophages, although the electrodes induced some increase of ROS, they did not increase the production of IL-6 cytokine. The non-invasive 3D stimulations system was tested with scaffolds that previously showed biocompatibility, pro-regenerative and angiogenic responses when implanted in rats. Impedance characterization of the scaffolds revealed that incorporating rGO significantly decreased their resistance across various frequencies in distilled water. However, in the presence of the cell medium EMEM, the higher electrical conductivity of EMEM dominates the impedance behaviour of the scaffold-medium systems. At a target frequency of 10 Hz, reported beneficial for neural regeneration, a scaffold impedance lower than 800 Ω was ensured. Low-frequency 3D ES significantly promoted neuronal differentiation, neuritogenesis, and increased neurite length—key cellular mechanisms essential for the formation of mature neuronal networks in neural stem cells. Moreover, we demonstrated that the stimulation was relevant even using non-conductive scaffolds, particularly in the ones composed only of a decellularized extracellular matrix. In future work, different stimulation parameters shall be used (*e.g.* higher signal frequencies or mixed patterns) and strategies can be combined simultaneously in different cell stages. The presented findings serve as a proof-of-concept for the effectiveness of non-invasive *in vitro* 3D ES across diverse scaffolds.

## Materials and methods

### GBI optimization and GBI-based electrodes production

Electrodes were prepared by Graphenest – Advanced nanotechnology. Briefly, graphene platelets were produced by a proprietary liquid-phase exfoliation process that comprises the utilization of ultrasonic cavitation and high-shear mixing. Resulting graphene platelets were characterized regarding morphology by scanning electron microscopy (FEI Quanta 650 FEG SEM, Portugal), with images acquired at 10 kV and under high vacuum using an Everhart Thornley secondary electron detector, low resistivity Si used as substrate, particle size distribution measured using *n* > 100 particles. Structural properties were assessed by Raman spectroscopy (Witec alpha300 R Confocal Raman Microscope equipped with a UHTS300 spectrometer coupled to the Andor Peltier cooled CCD detectors, Portugal) by signal acquisition performed with a 532 nm excitation line, 100× lens – NA = 0.9 – and 600 g mm^−1^ grating. Elemental composition and chemical state were assessed by X-ray photoelectron spectroscopy (XPS) (Thermo Scientific ESCALAB 250Xi, Portugal) through acquisitions under ultra-high vacuum, spectra generated by an Al monochromated X-ray source operated at 15 keV and 200 W power, and peak-fitting using Avantage software with smart-type background subtraction and charge correction to the reference value of adventitious carbon.

For the preparation of graphene-based ink (GBI), a liquid-phase ink was developed due to its versatility and potential for high-throughput and low-cost manufacturing techniques such as screen printing or inkjet printing. The prepared graphene platelets were dispersed in a green solvent, *i.e.* terpineol (mixed isomers, 98%, Alfa Aesar), aided by ethyl-cellulose (ethoxyl content 48%, 10 cps, Acros Organics) as a binder, at room temperature for 30 minutes. Ethyl-cellulose has previously demonstrated efficiency in dispersing graphene in various solvents while also allowing to control ink rheology.^52^ Several concentrations of graphene and binder were tested based on the qualitative evaluation of adhesion to the substrate, coverage quality and viscosity for efficient screen printing. The viscosity of GBI was determined by rheology (CP-2000 Plus Cone Plate System, Portugal, spindle 6005, RT, shear rate range 20–200 s^−1^).

Electrodes based on GBI were screen printed onto a polyethylene terephthalate (PET) foil with 100 μm thickness, using a custom metallic frame with a 1500 90-40Y screen mesh. The GBI was used to print the working (diameter = 8 mm) electrodes using 3 printing steps to avoid defects that could compromise their conductivity. The tracks for the GBI electrodes were printed in one step using a commercial silver ink (Henkel, Loctite EDAG PF-410 E&C), while a blue polymeric one was applied on top of the electrode channels as the dielectric element (Sun Chemical Gwent Electronic Materials, D50706P3), named here DI. After printing, the electrodes were cured in an oven at 100 °C for 30 minutes. Final sheet resistance was measured using a custom 4-tip probe and a sourcemeter (Keithley 2450, Portugal, operated in current bias mode – 2.1 V, 5.0 mA).

### GBI biocompatibility and immune response studies

RAW-264.7 macrophages were cultured in Dulbecco's Modified Eagle Medium (DMEM) supplemented with 10% (v/v) FBS, 1 mM l-glutamine, 800 μg mL^−1^ penicillin and 800 μg mL^−1^ streptomycin in a 5% CO_2_ humidified atmosphere at 37 °C for 24 hours in direct contact with graphene-based ink produced by Graphenest (GBI) and a commercial dielectric ink from Sun Chemical (DI). Control macrophages were cultured in parallel in the absence of these materials. Each experiment was carried out three times in triplicate. Mitochondrial activity of RAW-264.7 macrophages was quantified after all these treatments using the WST-8 test from Sigma-Aldrich (St. Louis, USA), according to the manufacturer's instructions.

The intracellular content of reactive oxygen species (ROS) in RAW-264.7 macrophages was measured by incubating the cells with 100 μM 2′,7′-dichlorofluorescein diacetate (DCFH/DA, Serva, Heidelberg, Germany) for 30 minutes after culturing the cells for 24 hours in direct contact with GBI and DI inks. Flow cytometry analysis was then performed using a FACScalibur Becton Dickinson flow cytometer, by exciting the sample at 488 nm and collecting the data with a 530/30 band pass filter. At least 10^4^ cells were analysed in each sample to ensure statistical significance.

The secretion of interleukin 6 (IL-6) was determined by enzyme-linked immunosorbent assay (ELISA, bioNova científica, Madrid, Spain), after culturing RAW-264.7 cells for 24 hours in direct contact with GBI and DI inks, according to the manufacturer's instructions.

For confocal microscopy studies, RAW-264.7 macrophages cultured for 24 hours in direct contact with GBI and DI inks were fixed with paraformaldehyde (3.7%) and permeated with Triton-X100 (0.1% in PBS). After 20 minutes of incubation with bovine serum albumin (BSA, 1% (w/v) in PBS), samples were stained with rhodamine–phalloidin (1 : 40), washed with PBS, and stained with 3 μM DAPI solution. Finally, samples were observed by using an Olympus Confocal Laser Scanning Microscope. Rhodamine fluorescence was excited at 546 nm and detected at 600–620 nm. DAPI fluorescence was excited at 405 nm and detected at 420–480 nm.

### Scaffold preparation and characterization

Two scaffold formulations were prepared as 3D microenvironments to probe the suitability of the 3D ES using the graphene-based electrodes. The scaffolds were manufactured based on an adipose-derived decellularized extracellular matrix (adECM) *via* ice templating. One formulation contained only the decellularized extracellular matrix (adECM scaffold) while the second combined reduced graphene oxide (rGO) and adECM in a 50/50 mass ratio (adECM/rGO). Both scaffold formulations were prepared according to the synthesis previously reported.^2^ Briefly, adECM was dissolved in 0.1 M acetic acid for two days and further crosslinked using 1-ethyl-3-(3-dimethylaminopropyl)carbodiimide (EDC, Sigma-Aldrich) and *N*-hydroxysuccinimide (NHS, Sigma-Aldrich) using 3.3 μmol of EDC per mg of adECM and an EDC : NHS ratio of 1. Crosslinking was left to occur for two hours prior to freezing and freeze-drying. For the adECM/rGO scaffolds, rGO was added after adECM dissolution and prior to cross-linking, and further dispersed *via* tip sonication. After freeze-drying, scaffolds were rehydrated by immersion in an ethanol solution followed by subsequent mixture of water:ethanol at the following ratios: 30 : 70, 50 : 50, 70 : 30, 80 : 20, 90 : 10 and 100 : 0. During the last immersion in sterile water, the scaffolds were also exposed to UV radiation for 45 minutes. Before cell seeding, the scaffolds were maintained in Eagle's Minimum Essential Medium (EMEM, Sigma-Aldrich) with 10% (v/v) fetal bovine serum (FBS, Sigma-Aldrich) and 1% (v/v) penicillin/streptomycin (P/S, Sigma Aldrich) for two hours to allow medium absorption.

### Neuroectodermal stem cells

A neural stem cell (NSC) line, specifically the NE-4C neuronal cell line obtained from ATCC (#CRL-2925), was used. The cells were seeded onto the adECM and adECM/rGO scaffolds, at a density of 75 × 10^4^ cells per scaffold in complete medium: Eagle's Minimum Essential Medium (EMEM, Sigma-Aldrich) with 10% (v/v) fetal bovine serum (FBS, Sigma-Aldrich) and 1% (v/v) penicillin/streptomycin (P/S, Sigma Aldrich).

### 3D electrical stimulation of NSCs

Electrical stimulation of NSCs seeded on the adECM-based microenvironments was investigated in 3D culture and its effects on NSCs proliferation and differentiation were evaluated. After NSCs seeding, the scaffolds were placed in the device wells and electrically stimulated every day for 1 hour, using a stimulation signal of 10 Hz, bipolar, with a current injection of 200 μA.

### Neural stem cell proliferation

The proliferation experiment was conducted for 7 days. The complete medium was changed every 48 hours while electrical stimulation was performed every day. On the first, fourth and seventh days, scaffolds were frozen to preserve the cells for DNA analysis. The DNA quantification relied on adding to each sample 1 mL of digestive papain solution at 60 °C for 16 hours. The digestive papain solution was prepared by adding 3.8 U papain per mL (Sigma-Aldrich) and 0.01 M l-cysteine (Sigma-Aldrich) to phosphate-buffered EDTA (PBE) buffer (0.1 M Na_2_HPO_4_, 0.01 M Na_2_EDTA, pH 6.5). After the 16 hours digestion, 50 μL of the digested solution was added to 50 μL of 1× TNE buffer (10 mM Tris, 1 mM EDTA, 200 mM NaCl, pH 7.4) and then added to a previously prepared 2 μg mL^−1^ Hoechst 33258 (Sigma-Aldrich) dye solution in 1× TNE. The fluorescence levels of the final digested solutions with dye were registered (Infinite 200 Pro, Tecan) at 348 nm and 456 nm and calibrated using Calf Thymus DNA standard (Sigma-Aldrich). The NSCs proliferation was also evaluated when in direct contact with the GBI electrodes for 11 days (Fig. S1). Proliferation was assessed *via* the resazurin assay as previously described.^3^

### Neural stem cell differentiation

The differentiation experiment lasted for 14 days. During the first 3 days of culture, the scaffolds were treated according to the proliferation protocol previously described. Differentiation was induced on the fourth day with 10^−6^ M all-trans retinoic acid (RA, Sigma-Aldrich) in a low-serum medium: EMEM with 1% (v/v) FBS and 1% (v/v) P/S. After 48 hours and every 48 hours that followed, the medium was replaced by a serum-free differentiation medium consisting of Dulbecco's Modified Eagle Medium/Nutrient Mixture F-12 Ham (Sigma-Aldrich) supplemented with 1% (v/v) B27 (Thermo Fisher Scientific), 1% (v/v) insulin–transferrin–selenium (ITS, Sigma-Aldrich) and 1% (v/v) P/S. Electrical stimulation was performed every day. The scaffolds were analysed by confocal microscopy combined with immunocytochemical staining as well as SDS-PAGE and immunoblot analysis.

### Immunocytochemical staining

Regarding the immunocytochemical staining, cells were fixed (1× fixation buffer diluted from a 10× stock solution consisting of 20% (w/v) formaldehyde, 2% (v/v) glutaraldehyde, 70.4 mM Na_2_HPO_4_, 14.7 mM KH_2_PO_4_, 1.37 M NaCl, and 26.8 mM KCl). Afterwards, they were permeabilized with 0.1% Triton X-100 (Fisher Scientific) in PBS for 5 minutes, blocked with 2% (wt/v) bovine serum albumin (BSA, Sigma-Aldrich) in PBS for 30 minutes, and then incubated for one hour and a half at RT with primary antibodies specific against β-tubulin III (mouse anti-Tuj1, Biolegend; 1 : 1000) and Glial Fibrillary Acidic Protein (chicken anti-GFAP, Sigma-Aldrich, 1 : 250). Then, the scaffolds were submersed for 45 minutes in DyLight488-conjugated anti-mouse (Invitrogen, 1 : 500) and Alexa Fluor 594-conjugated anti-chicken (Invitrogen; 1 : 1000), and counterstained with 3 μM DAPI (Sigma-Aldrich) for 15 minutes. Finally, they were mounted on glass microscope slides and observed in a LSM 810 confocal microscope (Carl Zeiss Microimaging GmbH, Jena, Germany). Cell differentiation was studied using the LAS X software on the confocal images to quantify the Tuj1 positively stained area with respect to the DAPI area. Neurite length was evaluated specifically on neurites branching out of cell clusters (*N* ≥ 30) using Fiji ImageJ software.

### SDS-PAGE and immunoblotting

For SDS-PAGE and immunoblot analysis, lysates were prepared by harvesting and finely mincing the scaffolds in a 1% Sodium Dodecyl Sulfate (SDS) solution, followed by sonication, and boiling for 10 minutes. After protein quantification using the Pierce™ BCA Protein Assay Kit (Pierce Biotechnology), lysates, normalized based on mass, were separated using a 5–20% gradient SDS-PAGE in Tris–glycine buffer and transferred onto nitrocellulose membranes. To assess protein loading in each lane, reversible incubation with Ponceau S (Sigma-Aldrich) was performed, followed by blocking with 5% non-fat dry milk in TBS-T for 2 hours at room temperature (RT). Subsequently, the scaffolds were incubated with primary antibodies in 3% BSA/TBS-T: mouse anti-MAP2 (Novus Biologicals NBP2-25156; 1 : 5000; 2 hours at RT and overnight at 4 °C). This was followed by incubation with the anti-mouse IgG secondary antibody (horseradish peroxidase-linked, Cell Signaling 7076S; 1 : 3000) for 1 hour at RT. Protein signals were visualized using enhanced chemiluminescence (Amersham ECL Prime or ECL Select, Cytiva RPN2236 and RPN2235) with a ChemiDoc Imaging System (Bio-Rad), and the results were analyzed using ImageLab Software (Bio-Rad). Protein quantifications were normalized to Ponceau S staining (Fig. S8). Statistical analyses were performed using One-way Anova or Kruskal–Wallis test.

## Author contributions

Conceptualization: PM, NB, MTP, BF and PAAPM; data curation: PM and BF; formal analysis: PM, NB, SV, BS, MC, LC, MJF, MTP; investigation: PM, NB, BS, MC, LC, BF; methodology: PM, GG, RS, LNA; resources: RDO, MTP, BF, RS, AL, PAAPM; validation: NB, SV, MJF, RDO, MTP, LNA, PAAPM, visualization: PM, NB, MTP, BF, software: GG, PF; supervision: NB, PF, PAAPM; writing – original draft: PM, NB, MTP; manuscript revision: NB, SV, BS, MTP, BF, AL, PF, PAAPM; funding acquisition & project administration: PAAPM.

## Conflicts of interest

There are no conflicts to declare.

## Supplementary Material

RA-015-D5RA02570B-s001

## Data Availability

All the relevant data that support this work are available in the article and SI. Supplementary information is available: the system specificities regarding the device characteristics: mode, amplitude range and resolution, maximum compliance, frequency range, no of channels, architecture of the electronic controller board, electrical characterization of silver ink tracks and GBI electrodes. See DOI: https://doi.org/10.1039/d5ra02570b.
