# Supplementary material for: Enabling 3D electrical stimulation of adipose-derived decellularized extracellular matrix and reduced graphene oxide scaffolds in vitro using graphene electrodes
Source: RSC Adv. 2025 Sep 1;15(38):31257–71. doi: 10.1039/d5ra02570b (PMC12400995; doi:10.1039/d5ra02570b)
Supplement: RA-015-D5RA02570B-s001 [file RA-015-D5RA02570B-s001.pdf]

Supplementary Information (SI) for RSC Advances.
This journal is © The Royal Society of Chemistry 2025

Enabling 3D electrical stimulation of adipose-derived decellularized extracellular matrix and reduced graphene oxide scaffolds *in vitro* using graphene electrodes

Patrícia Alexandra Martins [a,b,c,†], Nathalie Barroca * [a,†], Sandra I. Vieira [d], Bárbara M. de Sousa [d], Guilherme Gil [b,c], Mónica Cicuéndez [e], Laura Casarrubios [f], María José Feito [f], Rosalía Diez-Orejas [g], Maria Teresa Portolés [f,h], Bruno Figueiredo [i], Rui Silva [i], Andrea Garcia-Lizarribar [j], Pedro Fonseca [b,c], Luís Nero Alves [b,c] and Paula A. A. P. Marques * [a]

Table S1. GBI optimization for screen printing

**Solvent system**	**Graphene (wt. %)**	**Binder (ethyl-cellulose) (wt. %)**	**Observations**
Ethanol + Ethylene glycol	1.8	0	No adhesion
Ethanol + Terpineol	12.3	0	No adhesion, high viscosity
Ethanol + Terpineol	1.22	0.36	Insufficient adhesion
Ethanol + Terpineol	8.8	1.56	Good adhesion but low viscosity
Ethanol + Terpineol	18	1.41	Uneven coverage, probably due to rapid evaporation of ethanol
Terpineol	12	8	Good adhesion, appropriate viscosity for screen printing

GBI electrode response to temperature and light conditions

Tests under varying temperature and light conditions were performed to better understand the thermodynamic and photoresponsive behaviour of the graphene-based electrodes (Figure S1).

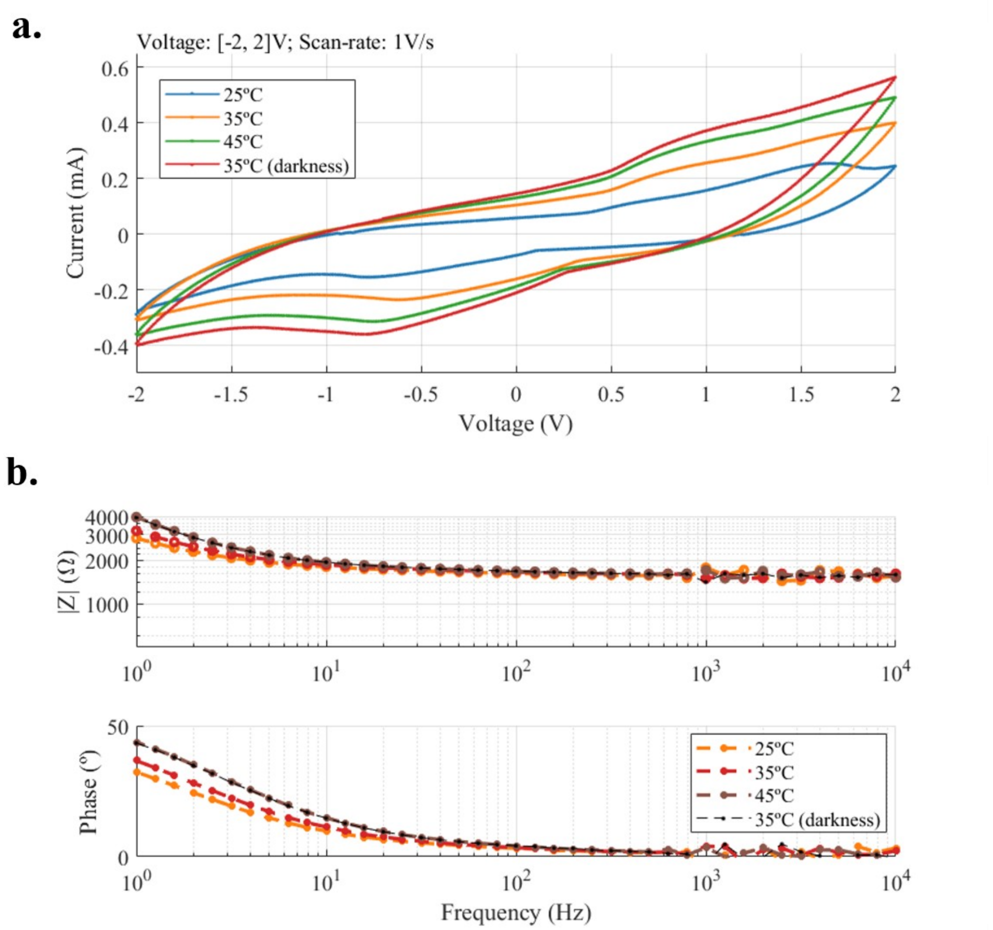

Figure S1. GBI electrodes response to variation of temperature and light conditions via a) Cyclic Voltammetry (CV) and b) Electrochemical Impedance Spectroscopy (EIS)

Interestingly, we observed that while CV measurements showed a decrease in impedance with increasing temperature, suggesting enhanced charge transfer and ionic mobility, EIS measurements under the same conditions indicated an increase in overall impedance. A similar divergence was seen under light exposure versus darkness. These differences likely stem from the distinct nature of each technique: CV emphasizes faradaic processes and rapid potential sweeps, while EIS probes steady-state impedance at low perturbation amplitudes, making it more sensitive to interfacial and dielectric changes. Therefore, while no direct heating of the system was observed during stimulation, these results point to subtle thermal and photo-induced changes in the electrochemical environment, potentially involving interactions at the electrode-electrolyte interface.

3D Electrical stimulation device – Specifications

For the 3D ES device, a wave generator was developed. The overall system comprises two main sections: i) the stimulation device and ii) a base station (computer). The stimulation device is based on a microcontroller architecture and is responsible for generating a set of customizable current square pulses while, at the same time, acquiring the electrical response of up to 16 scaffolds to such signals and transmitting them via wireless communications. The base station on the other hand includes a Graphical User Interface that allows users to set the properties of the electrical signals and view the scaffold voltage in real-time. All the samples are also stored in a database that can be accessed later for data processing via Matlab or other software. Table S2 presents the main characteristics of the 3D ES device:

Table S2. Stimulation Device characteristics

Mode	Amplitude and frequency - **current** controlled square pulses with duty-cycle control
Amplitude range	0.1 – 20 mA
Amplitude resolution	0.05 mA
Maximum compliance	20 – 25 V
Frequency	10 – 10 kHz
Nr. of channels	16

While both current and voltage are the primary controllable variables for electrically stimulating cells and tissues, range and characteristics vary significantly according on medium, materials, and dimensions. As a result, our 3D *in vitro* electrical stimulation system was designed with highly adaptable parameters that enable researchers to tailor the current parameter, specifically the controlled variable, to the system's characteristics.

Electronic controller board

The electronic controller board was designed to independently stimulate 3 groups of wells (i.e scaffolds) with different current signals and measure the voltage drop across each of the scaffolds. The system architecture, depicted in Figure S2, comprises three 3 main components: signal stimulation, voltage acquisition, and communications. The Unit Coordinator is responsible for managing all the communications (i.e., receiving user commands and transmitting the acquired data) and starting and stopping the stimulation process. The arbitrary pulse generator is only responsible for creating the desired electrical stimulation patterns. Finally, a high-performance external ADC is used to measure the voltage in each scaffold.

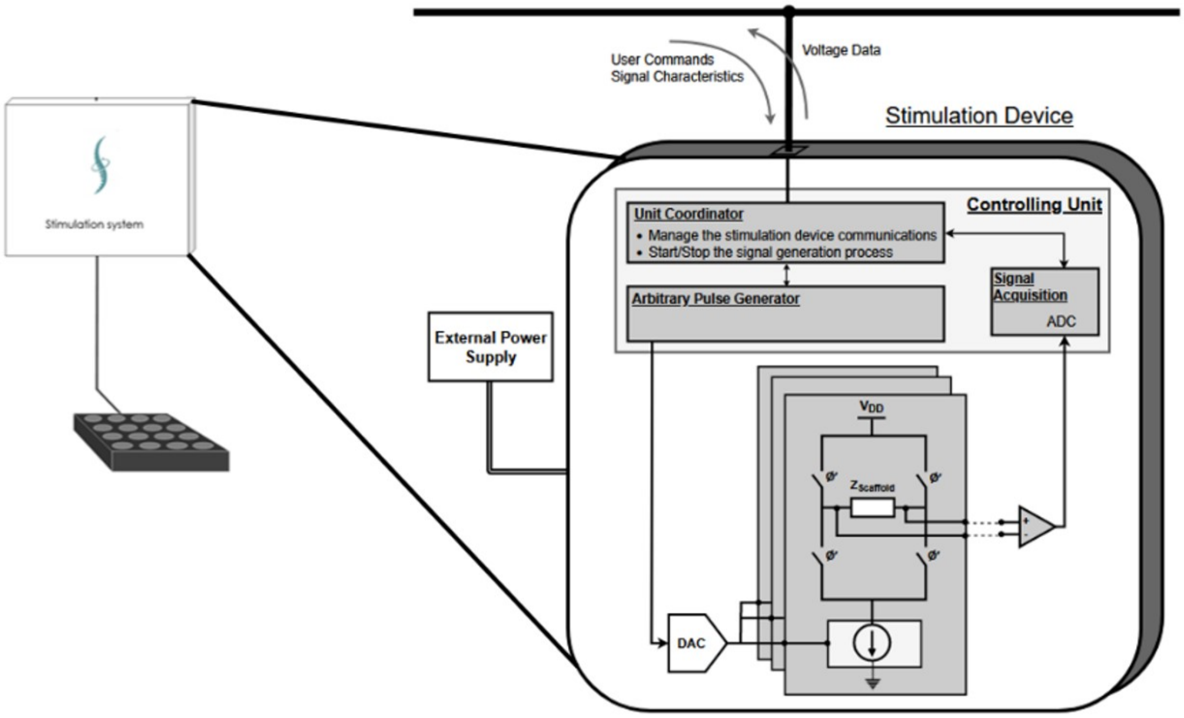

Figure S2. *In vitro* 3D ES architecture comprising three main components: signal stimulation, voltage acquisition and communications.

The Control Unit (Figure S3) is wirelessly connected to a computer (Wi-Fi). A C#-based application on the computer configures the stimulation characteristics (signal type, current values, and timings, for example) and initiates the stimulation process. Each pulse generator generates a single electrical signal, which is applied to 16 separate pairs of electrodes (bottom and top) via the analog front-end. The Signal Acquisition module samples the voltage across each of these sixteen pairs of electrodes and transmits it to the computer. The result is not three distinct stimulation devices, but rather a single device capable of simultaneously stimulating three distinct groups of scaffolds (3x16), enabling a more versatile approach to *in vitro* electrical stimulation research. The prior architecture with the three stimulation systems is represented in Figure S4. The device may create up to 25 square pulses with unique duty cycles

and frequencies ranging from 1 to 10 kHz, or constant DC pulses with a minimum frequency of 0.0001 Hz. The voltage readings in each scaffold are captured using an external ADC (MAX11131) and then supplied to the computer application over a serial interface.

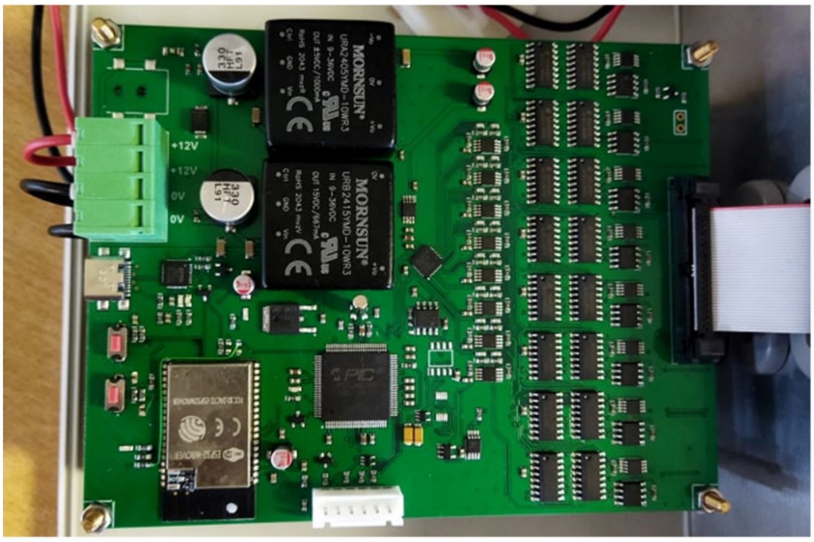

Figure S3. 3D ES control unit.

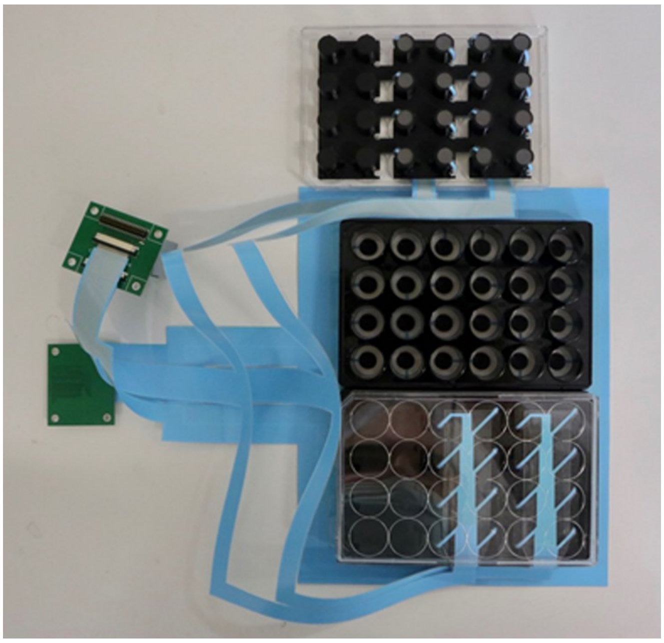

Figure S4. 3D electrical stimulation device: Two 24-well plates coupled with the 3 independent groups of 16 pair of electrodes (top and bottom).

With respect to the electrodes, their design comprises a multilayer system screen-printed on PET sheets (no surface treatment) with three 3 different inks: working electrodes made of the GBI ink, conductive tracks made of silver ink, and a blue dielectric ink to prevent short-circuits (the commercial ink from Sun Chemicals, named DI in the manuscript). Both the top and bottom electrodes are connected to the control unit described above and are ready to be in direct contact with the bottom and top surfaces of the scaffolds, as well as with the entire volume of the cell culture medium. Figure S6 presents the electrical characterization of the 48 silver ink tracks and of the "48 silver ink tracks + GBI electrodes" for the bottom electrode as a function of the track length between the control unit and the stimulation wells. The DC resistance (Ohm) was measured with a Keithley 2450 and two probes at a constant current of 200 mA and a voltage limit of 2 V (Figure S6b). 30 data points were taken with a delay of 1 s. As illustrated in Figure S6a, a longer track length to the stimulation wells results in a larger DC resistance in both situations (silver and silver + graphene). This measurement clearly demonstrates that graphene ink not only increases DC resistance (by an average of 200 Ohms) but also increases variability (R2 = 0.6742 versus R2 = 0.9806), which is mitigated by the Control Unit.

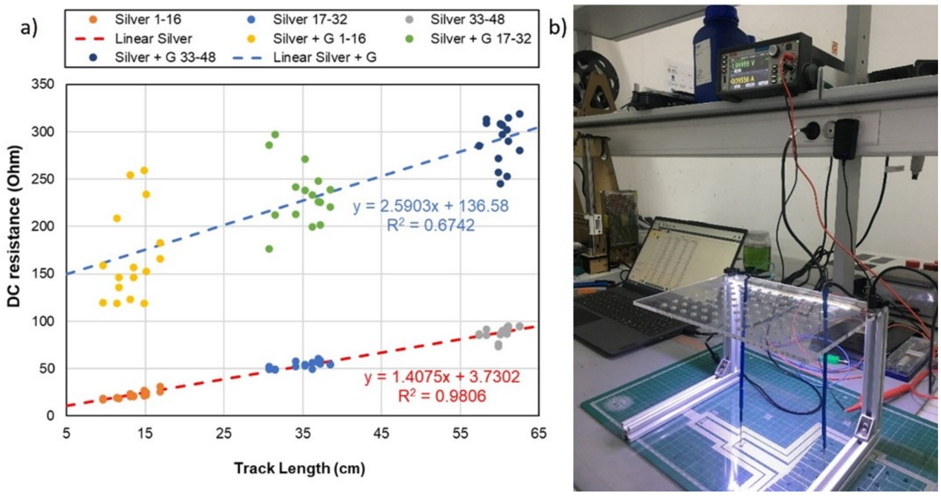

Figure S5. a) DC Resistance of the silver ink tracks and of the silver ink tracks plus the graphene dots electrode as function of the track length between the Control Unit and the stimulation well; and b) DC resistance measuring apparatus.

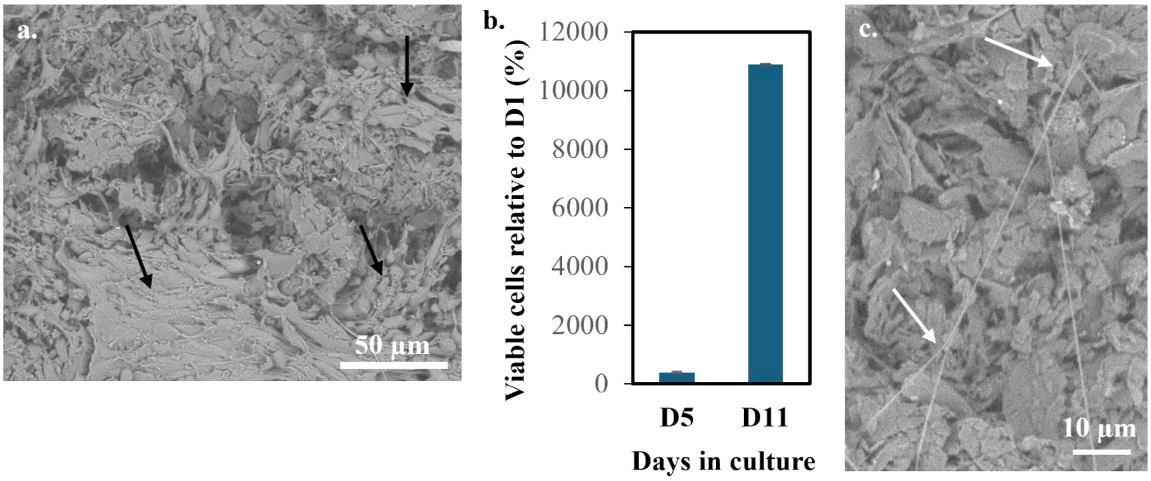

Figure S6. Neural stem cells culture in direct contact with GBI electrodes for 11 days: a) SEM micrograph showing large cell clusters; b) metabolic activity of NSCs and c) SEM micrograph showing neurite-like extensions.

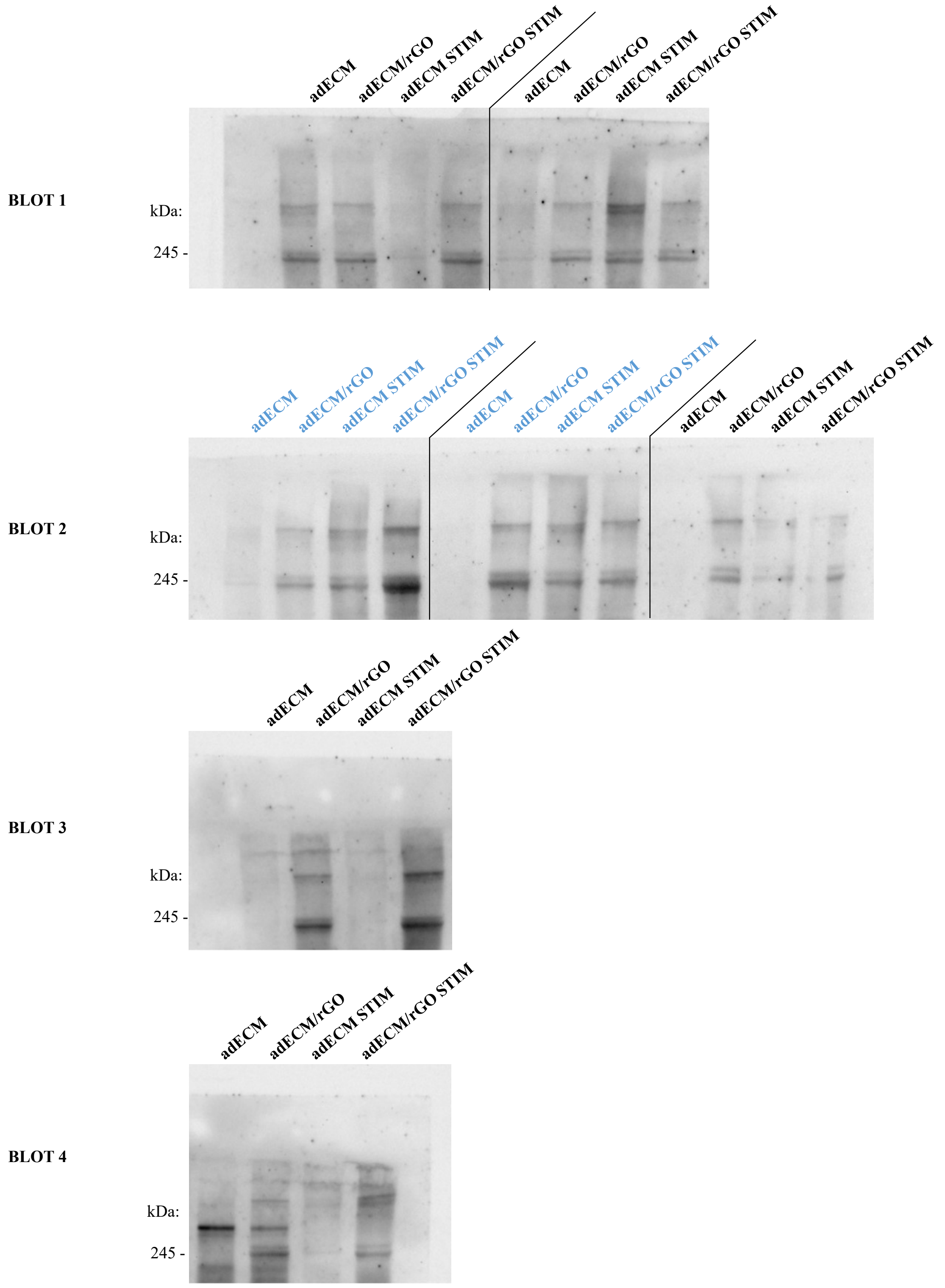

Figure S7. Raw images of all blots (7 biological replicas) used for the relative quantification of MAP2 levels in the immunoblot assays. The representative blots presented in Figure 7f (main manuscript) are highlighted in blue. Notably, there is some variability between replicas, likely due to differences in protein extraction efficiencies from each scaffold, in addition to the usual biological variability.

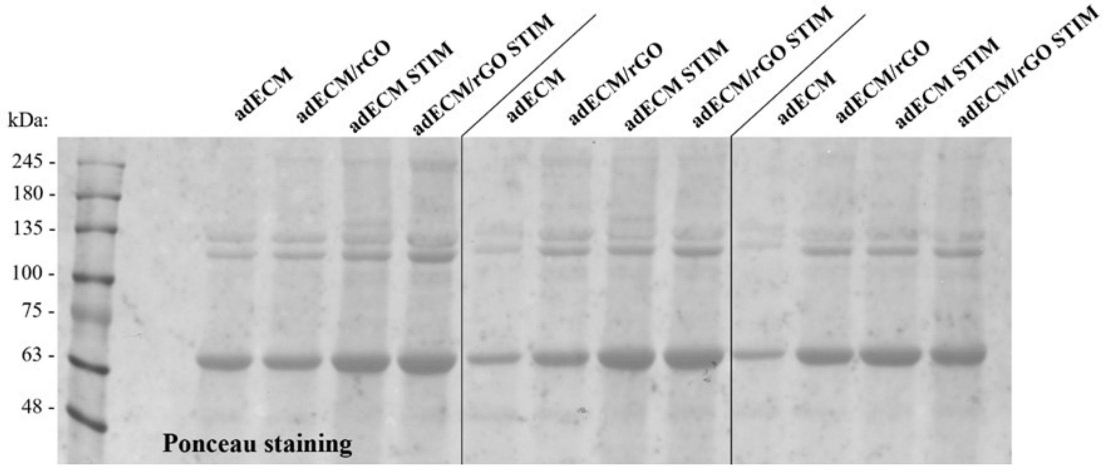

Figure S8. Membrane staining with loading controls. The nitrocellulose membranes used in the MAP2 immunoblot assays were stained with the Ponceau S dye, which binds to all proteins in the blot and is widely used as loading control.
